# Supplementary material for: Patterns of Intron Gain and Loss in Fungi
Source: PLoS Biol. 2004 Nov 30;2(12):e422. doi: 10.1371/journal.pbio.0020422 (PMC532390; doi:10.1371/journal.pbio.0020422)
Supplement: Table S1 — Also available at http://genes.mit.edu/NielsenEtAl/. (4.3 MB ZIP). [file pbio.0020422.st001.zip › NielsenEtAl/html/1195.html]

AN4460.1.NCU03628.1.MG08178.1.FG06788.1


```
 CLUSTAL W (1.82) Multiple Sequence Alignments - Introns Inserted


Sequence 1: NCU03628.1	922 aa
Sequence 2: FG06788.1	907 aa
Sequence 3: MG08178.1	938 aa
Sequence 4: AN4460.1	977 aa
Alignment Length: 998 aa
Number Identitical Residues: 349 aa
Alignment Score (without introns) 19002


MG08178.1 	MA-----FKTTFEPEKVIQPIFTGGSIALDSKAQILASTLGEDAVLTDITTGKRLATIEG
NCU03628.1	MAYRQTTTKTTFEVNNVIQPIFTGGSVALDNGARILATTLGEQAVLTELNTGKQLAEIEG
FG06788.1 	MANKQP-LKTTFDIKHVIRPIFTGGSVSIDNDARILATVLGEDVVLTDPTNGKHLAQIEG
AN4460.1  	MS--KAVVKTTFEASRTLRPIYTGGSTALDASGRLLVACVNEDALVVDLETGNQLATLDG
          	*:  ..  ****: ...::**:**** ::*  .::*.: :.*:.::.:  .*::** ::*

MG08178.1 	0DGEPISTLTL1TPSSSHLIISSRSLSLRIYSLAIDSDNA-SIEATLTRTLKPHGTPVVV
NCU03628.1	~DGEPISTLTI~TPSASHLIVCSRSLTMRIYSLKVSPDYD-SIEATLVRTTKPHATPVVV
FG06788.1 	0DGELISTLTL1TPSGSHLIICSRSLSMRIYSLKRSEDDE-TIEATATRSVKAHSTPVNV
AN4460.1  	0DGEVITSLAI1TPSASHVILCSRSMSMRIYSLSQYEDTTPTLETKLVRSLKPHTAPVVT
          	 *** *::*:: ***.**:*:.***:::*****    *  .::*:. .*: *.* :** .

MG08178.1 	LAVDRTSTLLATGAADGAIKVWDIVGGYVTHTFRGPSVLASALRFFEVAATAEDTKGQKK
NCU03628.1	LAVDRTSTLLATGAADGAIKIWDIVGGYVTHTVSGPSVLISALHFFEIAALEDQASN-KN
FG06788.1 	LTVDRTSTLLATGGTDGAVKVWDIVGGYVTHTFRGSSVLVSSLRFFEAASRSNETQSRKG
AN4460.1  	TATDSTGTLLATGAADGSIKVWDIRGGYITHSFHGHGGVISALCFFQPPTAEHDGKLSSK
          	 :.* *.******.:**::*:*** ***:**:. * . : *:* **: .:  .: .  . 

MG08178.1 	KKGKKSQ-----GAAEAESDVNSTTVN---FRLASGSQDGRVRVWDLHKR-------AAI
NCU03628.1	KKNRRKS-----TAEEEQAQDDSVASK---FRLAYGTQDGKIRIFDLSKRKTYPVYADPT
FG06788.1 	KKSKRQE-----ESDDE--EESSTTIN---FRLACGQQDGKVRVWDLHKR-------NCV
AN4460.1  	KKPKRKTGMAGDDDDNEMMDLDSASNSNGAFRLASGSEEGKVRVWDLNKK-------KSI
          	** ::. . :..   :   : .*.: ...:**** * ::*::*::** *:          

MG08178.1 	GDLESHVSDVTGIDYSAQENSLITASRDKTIIWWDVRTWQPK-KIVPSLELVEAAGFLDD
NCU03628.1	SRREAHESNVQSLDYSPELHALLSGSRDKTMTVWLWKEGTWRGTPMLRHEGVEAVGFLNN
FG06788.1 	ANLDSHVSDVQGLDYSPEQHALVTAGRDKTITWWDAKSWKIR-KVVPCLELVEAAGFIDD
AN4460.1  	ASLDSHVSVVRSLSYSLTENALLSAGRDKTVIVWDMRTFKTR-RIIPVLESAEAASFVAE
          	.  ::* * * .:.**   ::*::..****:  *  :    :   :   * .**..*: :

MG08178.1 	GRLAYSAGANGRVRLWESETGREVTKEQSPRTEGEAFVGAVYCAQQSLLVCVQSDHTLVL
NCU03628.1	GSLMYSAGTSGLLRLWDTTTHQEITAKQDAKSEAESIVSALALPHKNLVVCAQSDFTLAL
FG06788.1 	GNLTYSAGANGSLRIWDTDTGKEVTPQQSAKSEEEGIVSVIYRPGLPFILLVQVDHTLAL
AN4460.1  	GGLCLVGGEYGRLRVWDCNRGGEVTGDQEAAPEFEAIIAIHYTPGMSFAMTVHADQTLRL
          	* *   .*  * :*:*:     *:* .*.. .* *.::.    .   : : .: * ** *

MG08178.1 	YTTPTVNSATI-PELEPLSPTRRISGTHDDIIDLGYLLPDRSLMALATNSEEVRVVSVS-
NCU03628.1	YRTPSVADATK-KSTKPLEPFRRISGTHDEILDLVYLLPDQSLMALATNSEDIRLVSVAD
FG06788.1 	YRPPQKADAAS-LSAP--EPFRRISGTHDDIIDLGYLLPDRSMVALATNSEDVRIVSVVE
AN4460.1  	HSLEPLSNLKPGTTIDPLPVVRRISGNDDDIIDLAYVGPDRSMLALATNTESIRIVSVGP
          	:      .   .    .    *****..*:*:** *: **:*::*****:*.:*:***  

MG08178.1 	-------QEAEGSTYFGQDVTALKGHEDIVIALDTDWSGHWLATAAKDNTARLWRIDPAN
NCU03628.1	KQPQ-LGDEEEGAAYFGHDVALLKGHEDIVMSLDVDWSGHWVASGAKDNTARLWRVDPAN
FG06788.1 	TQDQDDGGNSGSSPYFGQDVALLKGHDEIVISLDIDWSGHWIATGAKDNTARLWRIDPAN
AN4460.1  	SDYR---PSTDGQEYFGADIAHLDGHDDIIICIDVDWSGHWLATGAKDNTARLWRLDPQN
          	..      .  .  *** *:: *.**::*::.:* ******:*:.**********:** *

MG08178.1 	NSFTCYATFTGHAESLGAIALPKSIPPKSSQAYTDPLNHPPSFLITGSQDLTVKKWEIAN
NCU03628.1	NSFECYAVFTGHIESVGAVALPKIVPPENSEAFKNPLDHPPAFLISGSQDRFVQKRDIPR
FG06788.1 	NSYTCWAVFSGHAESLGAVALPKSVPPESSAARTDPLSHPPAFLITGSQDQTVKKWEIPR
AN4460.1  	SSYTCFALLTGHAESLGAISFPRDSPPPDTPAYKAPVNHPPPFVITGSQDRTVKRWNVGK
          	.*: *:* ::** **:**:::*:  ** .: * . *:.***.*:*:****  *:: :: .

MG08178.1 	VA-QQGSK-KASRAAFTRKAHEKDINAVDISSTGQLFASASQDKVVKIWSVQEGEVQGIL
NCU03628.1	TS-QKGAV-STS---LRRLAHEKDINALDISPNGKLFASASQDKTVKIWDSATLEVVGIL
FG06788.1 	TA-QQGHK-NSARAIFTRKAHEKDINAINVHHSNQLFASASQDKTVKIWSAEEGEVQGIL
AN4460.1  	LGPTIGNKPHTPKALFTRKAHDKDINALDVSPSSALFASASQDRTVKIWSADDGSVVGVL
          	 ..  *  . :. : : * **:*****:::  .. ********:.****.    .* *:*

MG08178.1 	KGHRRGVWSVRFAPADMPILQGEDGATSAGKGIVLTGSGDKTIKIWSLTSYTCIRTFEGH
NCU03628.1	KGHRRGVWTVRFAPQGMPAIQGETGTAVSGKGVILTGSGDKTIKLWNLSDYTCLRTFEGH
FG06788.1 	RGHKRGVWSVQFSPAQLPAIQGEDG-PVTGKGVILTGSGDKTIKLWNLASYTCVRTFEGH
AN4460.1  	RGHKRGVWSARFSPKGTPILS-ADAKSSTNRGMIVTGSGDKTVKLWSLSDYSCLLTFEGH
          	:**:****:.:*:*   * :.   . . :.:*:::*******:*:*.*:.*:*: *****

MG08178.1 	SNSVLKVIWLPIP----STKEDNE--------AAKKRPVQFASAGGDGLVKVWDANTGEC
NCU03628.1	SHNVLKVVWLRLP----KAADDAEDEAAAAASKAKQR-IQFASAGADSLVKVWDANLGET
FG06788.1 	SNSILKVAWLNMP----TSQE-----------QSKKR-VHFASAGGDGLVKVWDANSGET
AN4460.1  	TNSVLKVLWLPPPDMSSKTEDEEDEDAAASKPNSIQLRPLVASAAADGLVKIWSPYTGEL
          	::.:*** **  *. ::.: :. ...:::: . : :    .***..*.***:*..  ** 

MG08178.1 	VATLDNHTDRVWALAVKPGTQGSIADADDVDMDGDSEEQVGDALVLASGSADSTITFWRD
NCU03628.1	ECTLDNHEDRLWTLTVHPKTN-----------------------MLVSGGSDSRVTFWKD
FG06788.1 	ECTLDNHEDRVWVVAVHPENN-----------------------TIVSGSGDSTVTFWKD
AN4460.1  	ETTLDNHTDRVWALASPTPSG-------TRDDVNSSATKSTSPYAIASGSADSTVTFWTD
          	  ***** **:*.::  . .          .  ..:  .  ..  :.**..** :*** *

MG08178.1 	TSSMTQLAAAKAADELVEKEQELQNHMRAGHYREAIVLALQLNHPGRLLSLFKTVMEEGS
NCU03628.1	TSAETHAAASSAALKLVEQEQQLENYIHAGAYRDAIILALQLNHPGRLLGIFTNVVTTNA
FG06788.1 	TSSETQAAASQAALKVIEQEQELENHIHAGSFREAITLALQLNHPGRLLSLFNSVMTTEK
AN4460.1  	TTSATYTAAVNANSARIEQDQQLQNYIVAGAYREAITLALQLNHPARLLSLFSAAIDAAD
          	*:: *  ** .*    :*::*:*:*:: ** :*:** ********.***.:*. .:    

MG08178.1 	NLATAEMRLDEDRKSLTGRAAVDEVIATLSDEQLSVLLLRLRDWNANARTAPVAQRILSV
NCU03628.1	----------PEEGSLCGIKAVDQVLGNLSDEQIFLLLLRLRDWNTNARTAPVAQRILWA
FG06788.1 	----------PDKGSLSGLRAVDEVLGSLSDEQIFLLLLRLRDWNTNARTAPVAQRILWT
AN4460.1  	DPHNTDNQGEDRANSFTGNPSIDEVLQTLDPANLYVLVLRLRDWNTNARHSKVAQRILFA
          	.  .:.   .    *: *  ::*:*: .*.  :: :*:*******:*** : ****** .

MG08178.1 	VVRSYPASRLAGLAVARRR-----AVGGKGVGEVLDALKAYTERHYRRLEELVDESYLVE
NCU03628.1	LVRLYPANKFSNLSVKG-------ARGQKSLKEVLNALRVYTERHYKRIEELVDESYLVE
FG06788.1 	LIRSYPASKFSNLSVKG-------AKGQKSLKDVLHAIRVYTERHYKRTEELVDESYLVE
AN4460.1  	LFRSYPASTFVELASSSIAKRNGDGRAAAAMKDILQALASYTERHYRRVEELEDESYLVE
          	:.* ***. :  *:     . .... .  .: ::*.*:  ******:* *** *******

MG08178.1 	YTLQEMDS--------LAPPVDMT--AIEEASRDVVMLET
NCU03628.1	YTLQEMDS--------LAPPAIEE----RDG--DLVMADA
FG06788.1 	YTLQEMDD--------LAPMLEDENALLQDGSEDVVMAG-
AN4460.1  	WVLGEMDGGIGLSALGAPTQGINEIEDKAEHGKDVVMLGA
          	:.* ***.. . :: . ..          : . *:**  :
```
